# Effect of filtration on morphine and particle content of injections prepared from slow-release oral morphine tablets

**DOI:** 10.1186/1477-7517-6-37

**Published:** 2009-12-22

**Authors:** Stuart McLean, Raimondo Bruno, Susan Brandon, Barbara de Graaff

**Affiliations:** 1 School of Pharmacy, University of Tasmania, Hobart, Tasmania, Australia; 2 School of Psychology, University of Tasmania, Hobart, Tasmania, Australia

## Abstract

**Background:**

Injections of mixtures prepared from crushed tablets contain insoluble particles which can cause embolisms and other complications. Although many particles can be removed by filtration, many injecting drug users do not filter due to availability, cost or performance of filters, and also due to concerns that some of the dose will be lost.

**Methods:**

Injection solutions were prepared from slow-release morphine tablets (MS Contin^®^) replicating methods used by injecting drug users. Contaminating particles were counted by microscopy and morphine content analysed by liquid chromatography before and after filtration.

**Results:**

Unfiltered tablet extracts contained tens of millions of particles with a range in sizes from < 5 μm to > 400 μm. Cigarette filters removed most of the larger particles (> 50 μm) but the smaller particles remained. Commercial syringe filters (0.45 and 0.22 μm) produced a dramatic reduction in particles but tended to block unless used after a cigarette filter. Morphine was retained by all filters but could be recovered by following the filtration with one or two 1 ml washes. The combined use of a cigarette filter then 0.22 μm filter, with rinses, enabled recovery of 90% of the extracted morphine in a solution which was essentially free of tablet-derived particles.

**Conclusions:**

Apart from overdose and addiction itself, the harmful consequences of injecting morphine tablets come from the insoluble particles from the tablets and microbial contamination. These harmful components can be substantially reduced by passing the injection through a sterilizing (0.22 μm) filter. To prevent the filter from blocking, a preliminary coarse filter (such as a cigarette filter) should be used first. The filters retain some of the dose, but this can be recovered by following filtration with one or two rinses with 1 ml water. Although filtration can reduce the non-pharmacological harmful consequences of injecting tablets, this remains an unsafe practice due to skin and environmental contamination by particles and microorganisms, and the risks of blood-borne infections from sharing injecting equipment.

## Background

It is common for many injecting drug users to prepare injections from tablets that are designed for oral administration [1,2]. Tablets contain therapeutically inactive ingredients to facilitate the lubrication, disintegration and dissolution of the dosage form [3]. These ingredients include talc, cornstarch, cellulose, magnesium stearate and waxes, which are not water soluble and their injection can cause complications. After injection into any blood vessel, particles will move downstream until they encounter a vessel too small to pass, where they lodge forming an embolism. Blockage of a vessel causes ischemic damage through a reduction in blood supply to the tissue downstream and can result in necrosis of the distal tissue. For example, injection of particles into a peripheral vein can lead to pulmonary granulomas, pulmonary oedema, emphysema and pulmonary fibrosis and hypertension [4,5]. Smaller particles (< 3-4 μm) can pass through capillaries and remain in the circulation until sequestered by the mononuclear phagocytic system, mainly in the liver and spleen [6]. Intra-arterial injection, whether deliberate or accidental, can impair blood supply to a limb and cause severe tissue ischemia and necrosis, leading to amputation [7].

Adverse reactions to illicit drug injections are commonly aggravated by infections due to the non-sterile methods of preparation and injection procedures. This is seen with local skin and soft tissue infections, which are the most common cause of hospital admissions of injecting drug users [8]. Filterable contaminants in drug injections contribute to many other cardiovascular and infectious complications [9,10].

Although appropriate syringe filters can remove particles from solutions for injection, their use has not become routine amongst injecting drug users. While there are many factors contributing to this, including cost, availability and performance of syringe filters, one substantial reason is the concern of many drug users that some of the drug would be lost in filtration.

The current study was designed to replicate preparation and filtration methods used by injecting drug users for injection of slow-release oral morphine tablets (MS Contin^®^, also known as MST Continus^® ^in some other countries). This was selected as injection of morphine tablets was reported as the last drug injected by 15% of the 2270 injecting drug users interviewed in the national 2008 Australian Needle and Syringe Program study [11] and as injected in the past six months by 47% of the 909 frequent injecting drug users interviewed nationally in the 2008 Illicit Drug Reporting System study [12]. MS Contin is the morphine tablet most commonly injected [12]. The current study aimed to compare the effectiveness of different types of commonly used filters on their ability to reduce particle content, and also their effect on amount of morphine remaining in the filtered solution.

## Methods

### Procedures used by injecting drug users

A survey of injecting drug users in the Hobart area (Tasmania, Australia) presenting to needle and syringe distribution outlets (n = 260) was conducted to determine the filtering methods they applied on their last occasion of morphine injection [13,14]. One-third (29%) had not used any filter; 41% used cigarette filters (34% hand-rolling cigarette filters); 21% used 0.22 μm syringe filters accessed through needle and syringe outlets. Minorities reported use of 0.45 μm syringe filters (2%), cotton balls (3%), combinations of cigarette and syringe filters (3%) or other makeshift filters (such as tampons, 1%). Injecting drug users in the Hobart area (n = 11) subsequently participated in detailed interviews to describe how they prepared injections from tablets and their filtration procedures. The methods used in this laboratory study are based on these methods.

### Extraction of morphine from tablets

One sustained-release 60 mg tablet of morphine sulfate (MS Contin^®^, Mundipharma, UK) was wiped with a sterile swab containing 70% isopropyl alcohol (Briemarpak, Briemar Nominees, Australia) to remove the orange coating (replicating the process used by injecting drug users), then placed on a glass petri dish to dry. The tablet was then extracted using either cold or hot Water for Injection BP (Pfizer, Australia). In the cold method, the tablet was thoroughly crushed using a glass mortar and pestle, and mixed with 3.0 ml water. The mixture was then stood for 5 min with occasional stirring. Injecting drug users employ a variety of improvised methods, such as crushing the tablet between two spoons. However, it was considered preferable to use a single, reproducible method in this study, although it is probably more efficient than the improvised methods and may result in fewer large particles.

The resulting drug extract was either kept for filtration, examined for particle content by light microscopy or analysed for morphine content by high performance liquid chromatography (HPLC). The mixture was transferred into a 5 ml plastic specimen tube for particle counting or a 50 ml volumetric flask for morphine analysis. The effect of different methods of filtration on the particle and morphine content of the extracts were also examined. Control solutions were prepared in an identical manner except that the tablet was omitted.

In the hot method of morphine extraction, the tablet was crushed by a porcelain pestle in a small (3 cm diameter, 4 cm high), round nickel crucible and the remnants adhering to the pestle were scraped into the crucible. Then 3.0 ml water were added and the crucible placed on a hot plate and gently boiled, with mixing, for 30-45 s by which time the mixture appeared to be clear and melted wax could be seen floating on top. The mixture was then stood on the bench until it felt cold to touch (about 5 min) before further treatment as described for the cold preparations.

It is important to note that cold extraction methods are recommended by Australian Intravenous Drug Consumer groups (Network Against Prohibition http://www.napnt.org/health/filtering_pills.htm; Safer Injecting Net http://www.saferinjecting.net/injecting-ms-contin.htm) due to a perception of loss of active dose following hot extraction techniques.

### Filtration

#### Cigarette type filter

A filter used for hand-rolled cigarettes (Ranch Slims, Stuart Alexander & Co., Australia) was cut down the side and the encircling paper removed. Following precisely the methods reported by injecting drug consumers, the filter was placed in the drug extraction mixture and moved around with the tip of a 5 ml syringe (Luer-Lok, sterile, BD Medical, Melbourne, Australia) until it became saturated with the liquid. The tip of the syringe was gently pressed against the side of the filter and the mixture slowly drawn up. This was repeated at two other sites on the filter, and the filter usually became blocked before all of the liquid was drawn up. A second filter was added to enable the filtration to continue until there was no mixture left.

As described for the unfiltered extract, the filtrate was kept for further filtration, particle counting or morphine analysis. Two successive 1 ml rinses of water were added to the mortar or crucible and, after moving the filter around in the liquid, taken up into the syringe and added to the first filtrate. With the hot preparation method, each rinse volume was briefly boiled then cooled before filtration. All containers were tared and weighed again at different stages to estimate the volumes collected. Despite the presence of tablet constituents, it was assumed that the specific gravity of all the mixtures was that of water to enable the volumes to be calculated. Aliquots of each rinse were taken for morphine analysis.

#### Cotton ball filter

A cotton ball (Home Brand Cotton Balls, Australia) was cut into four equal parts which were rolled into smaller balls. One small ball was placed in the drug extraction mixture and gently moved around with the tip of a 5 ml syringe to absorb the liquid. The mixture was taken up into the syringe and treated as described for the cigarette type filter.

#### Syringe filter (0.45 or 0.22 μm)

The mixture was taken up into a 5 ml syringe fitted with a 19G needle. The needle was removed and replaced by a 0.45 or 0.22 μm sterile syringe filter (33 mm diameter, Millex, Millipore, Ireland) and the syringe contents were slowly pushed through the filter. In some experiments the filter was flushed with a 1 ml rinse volume of water.

#### Combined filters

After initial filtration through a cigarette type filter, followed by two 1 ml rinses, the combined filtrates were taken up into a 5 ml syringe and then the needle was replaced with a 0.45 or 0.22 μm syringe filter and the syringe contents gently expelled into a container. The filter was flushed with another 1 ml water.

### Analysis of particle content

Glass microscope slides and coverslips were cleaned in laboratory detergent (Decon 90, Decon Lab., Sussex, UK), rinsed in tap water, distilled water and methanol, then dried in air. In some experiments an overnight acid wash (conc. nitric acid) was included before the water rinses.

A 20 μl aliquot of each sample was pipetted on to a slide and covered with a 22 × 22 mm coverslip. This volume filled the area under the coverslip. The slides were viewed under a light microscope (Gillet and Sibert, London, UK) in transmission mode. The eyepieces (Leitz Wetzlar, Germany) had reticules which enabled particles of different sizes to be counted. One eyepiece showed a rectangular area (360 × 250 μm with 20× objective) and the other a linear scale (0-10 by 0.1 unit) whose length was 400 μm with 20× objective. The rectangle and scale were calibrated using the ruled lines of a Neubauer blood cell counting chamber (Hawksley, London, UK). Thus the smallest particle that could be measured with the 20× objective was 4 μm (0.1 on the scale) although smaller particles could be seen and counted. For each sample, five fields were chosen for counting, at the four corners and centre of the coverslip, selected by moving the microscope stage without looking through the eyepiece. Particles were counted if they were inside the rectangle, or overlapped the bottom or right side, but not if they overlapped the top or left side.

Particles were counted in size groups, using the scale and a factor based on the magnification of the objective to estimate size. For example, particles 50-99 μm were counted with the 5× objective, and corresponded to scale readings 0.3-0.5. Particles 20-49.9 μm were counted with the 20× objective and corresponded to scale readings 0.5-1.24. Clearly this level of precision was not possible, and borderline particles could have been assigned to either of two adjacent groups. Total particles in each size group were estimated from the average count per field multiplied by the ratio of field area/coverslip area (= number of particles in 20 μl), then by the ratio of the volume of the injection (ml)/0.02 ml to give the number of particles in the injection mixture. Counts are given as mean ± SD of three replicate preparations. Photomicrographs of slides were taken with an Olympus BX50 microscope fitted with an Olympus DP50 digital camera.

### Analysis of morphine content

The tablet extract or its filtrate was made to volume (50 ml) with 0.5% acetic acid. About 5 ml was filtered through a syringe filter consisting of a nylon prefilter and 0.45 μm filter (Chromacol, UK). This step was required to remove particles before HPLC analysis. The relatively large volume and acidic pH was expected to maximise the dissolution of any morphine that remained in the particles, and to minimise any retention by the filter. An aliquot of this filtrate was diluted ten-fold and analysed, in duplicate, by HPLC using a Varian 9010 instrument (Varian, Australia), a C18 reversed-phase column and UV detection at 286 nm. The mobile phase was 90% phosphate buffer (50 mM, pH 3) and 10% methanol, and flow rate 0.7 ml/min. Injections were made using a 20 μl loop. Standard solutions were prepared from morphine sulfate BP (British Drug Houses, London, UK) and there was no internal standard. The retention time of morphine was 4.2 min. Calibration curves from 2.5 - 200 μg/ml were prepared each day and linearity was excellent (r^2 ^> 0.99). As the morphine content of the tablets is given in mg morphine sulfate, we have done the same with the morphine recovered from the tablets.

## Results

### Cold extraction, unfiltered

The outer coating was readily removed with an alcohol swab and the tablet crushed easily although continued grinding did not result in an ever-finer powder since the waxy nature of the powder resulted in some re-compaction.

**Figure 1:**
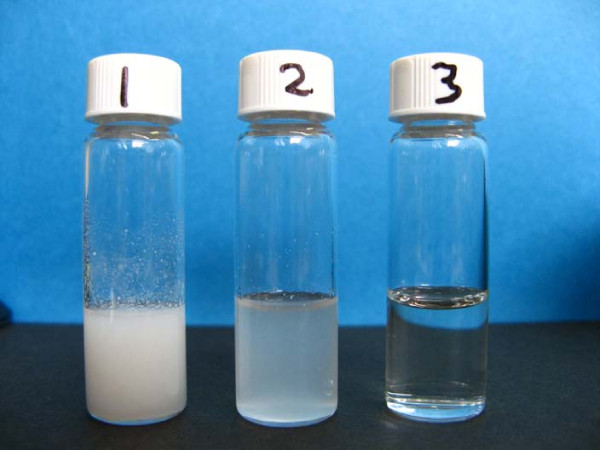
**Injection mixtures (cold extraction)**. Each mixture was prepared from one tablet as described in Methods. 1, unfiltered; 2, cigarette filtrate; 3, cigarette then 0.45 μm filtrate.

**Figure 2:**
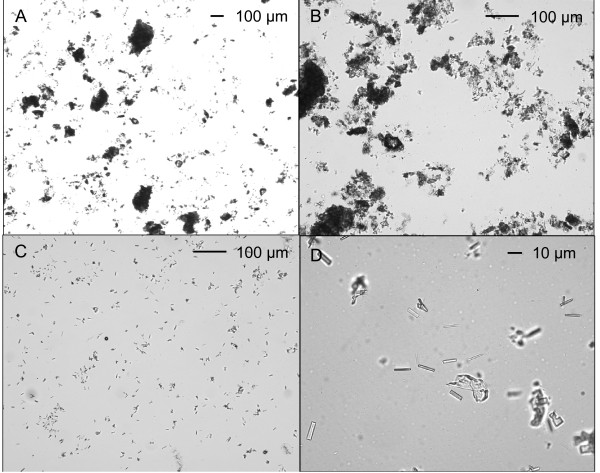
**Particles in unfiltered mixture (cold extraction)**. Photomicrographs of particles from a single preparation.

The crushed tablet suspended in cold water gave an opaque suspension with a milky appearance (Figure 1). Macroscopic particles were evident on the wall of the tube. The mean morphine content (as morphine sulfate) was 56 ± 2 mg from 60 mg tablets (Table 1). Microscopic examination showed many particles ranging in size from > 400 μm to < 5 μm (Figure 2A). Particles were not uniformly distributed across the coverslip area. Most of the fields counted were crowded and heterogeneous, making it difficult to count every particle, and the fields were usually dissimilar. The larger particles (> 50 μm) tended to be amorphous in shape and to some extent appeared to be agglomerations of smaller particles (Figure 2B). In counting the particles, the size group was taken to be that of the agglomeration, regardless of how friable it appeared. Thus the assignment of particles to size categories was based on a subjective judgement of whether they appeared to be separate or joined. Small particles (< 5-10 μm) sometimes drifted across the field, and had to be counted quickly while they remained in view. However, some particles would have been missed. The smallest particles (< 5 μm) were generally too numerous to count accurately, and their counts represent a minimum number.

**Table 1:** Recovery of morphine after cold extraction and filtration

Filtration	Volume of extract (ml)	Amount of morphine (mg)	**P versus cig**. filter alone*
**None**	3.0	55.8 ± 1.9 (N = 4)	

**Cigarette filter**			
First filtrate	1.7 ± 0.1	31.7 ± 2.3	
First rinse	1.1 ± 0.0	12.8 ± 0.7	
Second rinse	1.1 ± 0.1	5.9 ± 0.2	
Total	3.9 ± 0.1	50.5 ± 1.8	

**Cotton wool**			
First filtrate	1.7 ± 0.1	30.5 ± 1.9	
First rinse	1.1 ± 0.1	10.6 ± 1.2	
Second rinse	1.0 ± 0.0	5.0 ± 0.8	
Total	3.8 ± 0.0	46.1 ± 0.1	0.099

**0.45 μm filter**	1.9 ± 0.0	33.9 ± 1.0	0.098^†^

**Combined filtration**			
Cig.+ 0.45 μm filter	5.0 ± 0.2	51.5 ± 0.7	0.701
Cig. + 0.22 μm filter	4.9 ± 0.2	51.9 ± 1.4	0.410

Data are mean ± SD, N = 3 except where otherwise indicated.

*This figure provides a comparison of the amount of morphine recovered through use of the target filter to that recovered through use of a cigarette filter. Comparisons were made by Mann-Whitney U test (a non-parametric analogue of the t-test), 2-tailed. Results are consistent with parametric analysis with Games-Howell post-hoc tests with the exception of ^†^0.45 μm filters (p < 0.05). P values greater than 0.05 suggest that it is unlikely that there is any real difference between the amount of morphine recovered in the two compared techniques.

In some preparations regular crystalline shapes were seen, with sizes ranging from rectangles of 10 μm × 25 μm, to rods of 1-2 μm × 5-10 μm (Figure 2C and 2D). Treatment with weak acid (0.5% acetic acid) dissolved the crystals. This, together with their shape, and the absence of other likely candidates in the tablet formulation, suggested that the crystals were morphine hydrate, which precipitated because of the alkalinity of the glass. This was confirmed by examining an extract which gave abundant crystals on the standard glass slides. The same extract gave no crystals on either glass slides which had been soaked overnight in nitric acid, or plastic slides. Because their formation was considered to be artifactual, the crystals were not included in the particle count. All slides were examined when freshly-prepared as crystal formation increased with time on the slide.

A solution of morphine sulfate 60 mg in 3 ml Water for Injection had a pH of 4.6. An aliquot (50 μl) was mixed with an equal volume of 50 mM NaOH, resulting in the formation of masses of small rods of the same appearance as those found in the tablet extracts. The cold unfiltered tablet mixture had a pH of 6.4, which was unchanged after filtration through the cigarette filter or 0.45 μm or 0.22 μm filters.

**Figure 3:**
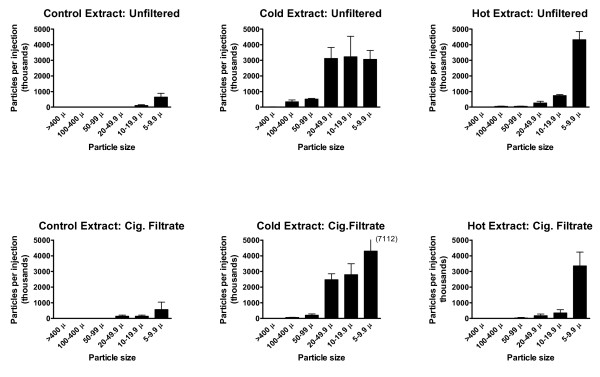
**Particles 5 μm or larger in injection mixtures**. Numbers of particles (in thousands) estimated to be in the total injection volumes, prepared without a tablet (control) or with cold or hot extraction. Upper panel, unfiltered; lower panel, cigarette filtrate. Total injection volumes are given in Table 1. Values are mean ± SD (N = 3).

**Figure 4:**
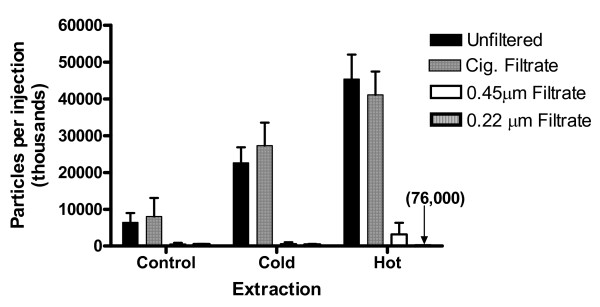
**Particles less than 5 μm in injection mixtures**. Numbers of particles (in thousands) estimated to be in the total injection volumes. The unfiltered extracts were passed successively through a cigarette filter then a syringe filter (0.45 μm or 0.22 μm). Values are mean ± SD (N = 3).

Figure 3 shows the number of particles sized 5 μm and larger in the total injection volume for three preparations: control (no tablet), cold extraction and hot extraction. Figure 4 shows the additional particles in the smallest size group (< 5 μm) of the same preparations, plotted separately because their counts were an order of magnitude greater than for the larger particles. The upper panel of Figure 3 shows the particle counts in unfiltered preparations. Even in the absence of a crushed tablet, some small (up to 20 μm) particles were found (Control: Unfiltered), reflecting contamination from the local environment. The unfiltered cold tablet extract produced a much larger number of particles of all sizes, with the numbers tending to increase as the size became smaller (Figure 3, Cold: Unfiltered). Although not apparent in Figure 3, there were also significant numbers of particles in the largest size group (> 400 μm), 12,000 ± 14,000 in the 3 ml injection volume. The large SD shows the inherent variability of the particle counts.

### Cold extraction, filtered

Two cigarette filters were required to enable the mixture to be taken into a syringe without the filter being blocked. The filtrate was milky, like the unfiltered mixture, but it was also more translucent and there were fewer particles on the wall of the tube (Figure 1). Only about half (1.7 ml) of the 3 ml water used to prepare the mixture could be recovered, with the remaining liquid remaining in the wet filters (Table 1). The mean morphine content of the filtrate was 32 mg, which is the amount expected to be in this fraction (1.7/3.0) of the total volume of extract. This indicates that the morphine was not binding to the filter, but was being retained because some of the solution was held up in the filter. Two successive 1 ml rinses with water recovered most of the remaining morphine (Table 1).

**Figure 5:**
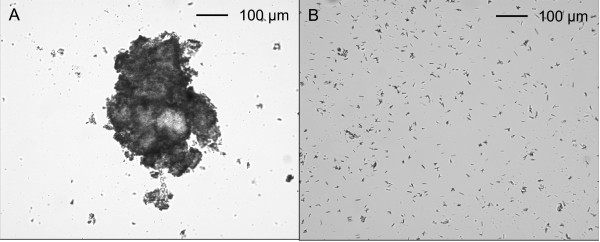
**Particles in cigarette filtrate (cold extraction)**. Photomicrographs of particles from a single preparation.

Some large particles escaped this filtration (Figure 5A), and there were large numbers of smaller particles and crystals (Figure 5B). The cigarette filter produced a large reduction (60-80%) in numbers of particles > 50 μm, a smaller reduction (10-20%) in particles sized 10-50 μm, and an increase (20-40%) in the number of smaller particles (< 10 μm; Figures 3 and 4).

The cotton ball filter gave a similar result to the cigarette filter: a milky filtrate with limited removal of particles (data not shown). However, the recovery of morphine may have been slightly lower (Table 1). Also, it was difficult to consistently reproduce the size and density of the filters.

The 0.45 μm syringe filter gave a clear solution (Figure 1), but the filter tended to block and it required considerable pressure to deliver the last of the filtrate. Additionally, the volume of filtrate was low (1.9 ml), containing only 34 mg morphine. However, this was the amount expected for the fraction of mixture which was filtered. The filtrate was relatively particle-free, and this will be described under the combination filtration procedure.

### Hot extraction, unfiltered

**Figure 6:**
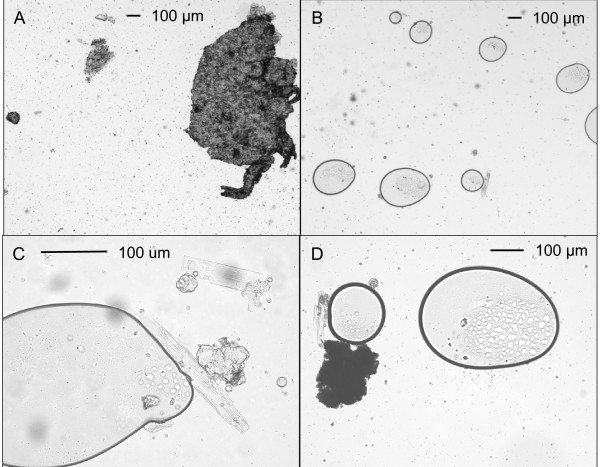
**Particles in unfiltered mixture (hot extraction)**. Photomicrographs of particles from a single preparation.

The tablet could only be coarsely crushed in the crucible, but everything melted or went into solution when the water was boiled. Care was taken to minimise evaporative water loss, since this tended to increase crystal formation. On cooling, the mixture became turbid and some large waxy solids separated. The largest masses were not taken up into the syringe and were therefore not included in the particle count. The aspirated mixture had a milky appearance, similar to that produced by cold extraction. The morphine extraction (59 ± 1 mg, Table 2) was virtually complete. Microscopic examination showed that, compared to cold extraction, there were fewer particles sized 10 μm or larger, and more sized less than 10 μm (Figures 3 and 4). However, the injection still contained an average 50,000 particles in each of the size groups 50-99 μm and > 100 μm. Figure 6A shows a field with one very large particle (> 400 μm) and many smaller particles, and panel B shows what appear to be solidified droplets of melted wax. The microscopic appearance of hot extractions was characterised by droplets, particles, and crystals (Figure 6A and 6D). The larger droplets had inclusions, either particles or other droplets. The formation of many small droplets contributed to the large number of small particles present.

**Table 2:** Recovery of morphine after hot extraction and filtration

Filtration	Volume of extract (ml)	Amount of morphine (mg)	**P versus cig**. filter alone*
**None**	3.0	58.9 ± 1.3	

**Cigarette filter**			
First filtrate	2.1 ± 0.1	42.4 ± 0.9	
First rinse	1.0 ± 0.1	9.1 ± 1.9	
Second rinse	1.1 ± 0.1	2.7 ± 0.1	
Total	4.1 ± 0.1	54.3 ± 2.6	

**Cotton wool**			
First filtrate	1.6 ± 0.2	35.3 ± 4.4	
First rinse	1.1 ± 0.0	12.1 ± 2.3	
Second rinse	1.0 ± 0.2	5.2 ± 0.6	
Total	3.8 ± 0.3	52.6 ± 3.1	0.393

**0.45 μm filter**			
First filtrate	1.9 ± 0.2	41.2 ± 2.3	
Rinse	0.9 ± 0.1	10.7 ± 2.4	
Total	2.8 ± 0.2	51.8 ± 1.9	0.203

**Combined filtration**			
Cig.+ 0.45 μm filter	4.5 ± 0.3	52.4 ± 1.6	0.400
Cig. + 0.22 μm filter	4.3 ± 0.6	49.6 ± 2.0	0.096

Data are mean ± SD, N = 3.

*Comparison of morphine recovery by Mann-Whitney U test (a non-parametric analogue of the t-test), 2-tailed. Results are consistent with parametric analysis with Games-Howell post-hoc tests.

### Hot extraction, filtered

Aspiration through a cigarette filter removed all of the largest particles (> 100 μm) but only 10-50% of the smaller particles (Figures 3 and 4). In preliminary experiments it was found that, if the mixture was still warm, many more of the wax droplets passed through the filter. As with the cold preparation, nearly all of the dose could be recovered after 2 × 1 ml rinses with water (Table 2).

Filtration through cotton wool balls gave no better recovery of morphine (Table 2) and, because of the variability in forming these filters, was not further considered.

The 0.45 μm syringe filter again gave a clear solution with recovery of 41 mg morphine, which increased to 52 mg after a 1 ml rinse (Table 1). The particle count is described after combination filtration.

### Combination filtration

**Figure 7:**
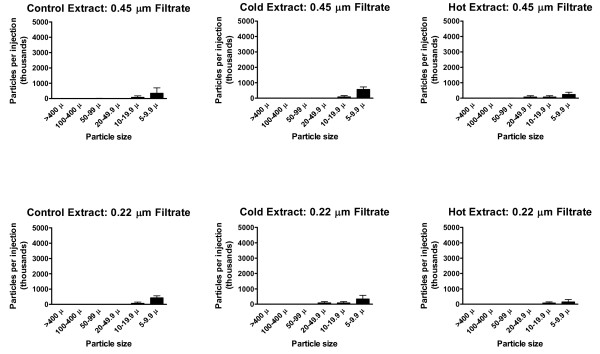
**Particles 5 μm or larger in syringe filtrates**. Numbers of particles (in thousands) estimated to be in the total injection volumes, prepared without a tablet (control) or with cold or hot extraction. Upper panel, 0.45 filtrate; lower panel, 0.22 μm filtrate. Total injection volumes are given in Table 1. Values are mean ± SD (N = 3).

Fresh extracts were prepared using cold and hot water and passed sequentially through a cigarette filter then syringe filter (0.45 μm or 0.22 μm), with rinses. After both cold and hot extraction with cigarette filtration, the subsequent syringe filtration step did not significantly reduce the recovery of morphine (Tables 1 and 2). Both syringe filters greatly reduced the particle count, to levels at or below the control counts (Figures 7 and 4). In some samples (e.g. 0.45 μm filtrate after hot extraction) there appeared to be large numbers of small (< 5 μm) particles or droplets. However, this will require confirmation by re-investigation using cleaner conditions.

## Discussion

Morphine in the MS Contin^® ^slow-release tablet is embedded in a complex dual matrix of hydroxyethyl cellulose and cetostearyl alcohol, designed to release the drug over 12 h [15]. Crushing the tablet disrupts this matrix, allowing the rapid extraction of morphine. The amount of morphine in prolonged-release tablets is permitted to vary by 5% [16] so a 60 mg tablet could contain from 57 to 63 mg morphine sulfate. The extraction of morphine by cold water (56 mg) and hot water (59 mg) was therefore essentially complete. None of the filters bound morphine, and their retention of morphine was due to the volume of liquid which remained. Consequently, rinsing the filters increased the recovery of morphine. Repeated rinses brought diminishing recoveries of morphine, and increased the volume to be injected, and therefore the number of rinses used in the combined filtration method was a minimized but nevertheless gave a good recovery (84-93%) of the extracted morphine. Overall, the extraction of morphine and its recovery after filtration was similar after cold and hot extraction.

The MS Contin^® ^tablet contains a number of constituents with low or no water solubility which are liable to produce particles in the extract [17]. These include cetostearyl alcohol, which is a mixture of two waxes: cetyl alcohol (1-hexadecanol, mp 49°C) and stearyl alcohol (1-octadecanol, mp 61°C); magnesium stearate (mp 88°C); talc, a hydrated magnesium silicate; and hydroxyethylcellulose (a gelling agent which is insoluble in water). The coating contains other insolubles such as iron oxide, but this was usually removed in preparing the extracts.

There are advantages and disadvantages in counting particles by microscopy rather than an instrumental method, such as the Coulter Multisizer which has been used to study the effectiveness of filters for heroin injections [18]. In this latter study, the instrument required considerable dilution of the sample (50 μl to 75 ml) with the possibility of dissolution of some particles which would have been present in the smaller volume to be injected. The dilution was made with an electrolyte (saline) which may also have affected particle solubility or aggregation. Microscopy avoided dilution and enabled examination of the appearance of particles, which gave insights into their origin (such as crushed solids, condensed wax droplets, and crystallised morphine) which in turn can indicate how they could be removed. However, microscopy necessarily examines only a small part of the total sample, adding to errors as discussed below.

Counting particles, especially in the unfiltered preparations, was inherently variable due to the large amount of insoluble material and its complex physical form. This variability also affected instrumental counting, and Scott [18] considered that variability in the particle counts made the exact values meaningless although useful for comparison of filters. In our study counts are presented as the number of particles in an injection volume in order to relate the data to health impacts. This required a large multiplier factor. For example a count of one 100 μm particle in 5 fields using the 5× objective would give an estimated 8,897 particles in the 3 ml dose volume, and one 10 μm particle (20× objective) would give 161,333 particles in 3 ml. If there were no similar particles in the other two replicate mixtures, then the mean particle counts would be 2966 for the 100 μm particle and 53778 for the 10 μm particle. The particle counts are therefore reported in thousands to avoid implying a level of precision which would be misleading. The counts are indicative estimates rather than precise determinations, but are nevertheless able to show that filtering can greatly reduce the number of particles injected.

The working area for preparing the injections was neither sterile nor particle-free, since the aim was to reproduce the typical conditions used for illicit preparations by injecting drug users. Consequently, a significant number of particles and fibres were found when control injections were prepared, showing that particles are ubiquitous unless removed by specific cleaning procedures. Fibres, however, were not counted as particles since they were present on control slides and were not considered to be tablet-derived. Environmental particles will vary widely according to local conditions but will add to the total particle burden in the injection. Not using a clean workplace became a limitation in counting particles in the cigarette plus 0.22 μm filtrate, in which virtually all tablet-derived particles had been removed.

The form of the waxes was evidently altered after melting and re-solidifying, with the formation of wax droplets of various sizes. Hot extraction resulted in a shift in particle size distribution, with the formation of more small particles (< 5 μm) and fewer larger particles. However, the remaining particle burden in unfiltered preparations was still too large for this to be considered other than harmful to inject. Pharmaceutical standards require that, measured by microscopy, injections of less than 100 ml must have, in total, no more than 3000 particles > 10 μm and no more than 300 particles > 25 μm [16]. The hot unfiltered morphine tablet preparations had, in the total volume of 3 ml, an average 1.1 million particles > 10 μm and 368,000 particles > 20 μm. For the cold preparations, the numbers of particles were 7.2 million > 10 μm, and 4.0 million > 20 μm.

The aqueous solubility of morphine is critically dependent on its ionization and therefore the pH, as the ionized form is freely soluble and the free base has a low water solubility (0.25 mg/ml at 35°C) [19]. These authors found that, at 35°C, the solubility of morphine in water was 13.39 mg/ml at pH 6.35 and 5.75 mg/ml at pH 6.69. This change in solubility with pH could be explained by the change in ionization and the low free base solubility. A 60 mg tablet of morphine sulfate (MW = 758.9) contains 45.1 mg morphine (MW = 285.3), or 15.0 mg/ml in the 3 ml extract. Using the amount of morphine sulfate recovered in cold extracts, this concentration would be 14.0 mg/ml. In either case, the concentration of morphine in the 3 ml extract will be critically close to, or exceed, its solubility, especially as the pH is slightly higher (6.4) and the temperature was considerably lower (about 20°C). From the p*Ka *of morphine (8.08) and the buffer equation, pH = p*K*_*a *_+ log_10_([base form]/[acid form]), it can be calculated that morphine is 1.8% unionized at pH 6.35 and 2.1% unionized at pH 6.40.

It was considered that morphine crystal formation was an artifact due to alkalinity in the glass microscope slide or cover, since they did not form on acid-washed glass or plastic slides. However, there is a significant risk of formation of morphine crystals in the tablet extracts,, and conditions of preparation could reduce this problem by decreasing pH or, preferably, increasing the volume of water. Although morphine will eventually dissolve in blood this will take some time, and any crystals which remain undissolved during the brief transit time from injection site to capillary bed are liable to cause embolisms.

The unfiltered tablet extracts must be considered extremely harmful as they contained many particles of all sizes. After intravenous injection, particles will flow through ever-widening vessels back to the heart and then they will enter the pulmonary circulation, where the smaller arteries which are 300-400 μm diameter [20] could be occluded by the largest particles found in unfiltered mixtures. Arterioles (9-40 μm diameter) and capillaries (7-9 μm diameter) could be blocked by the smaller particles. Even particles too small to embolize may cause vascular injury. Small airborne particles (< 2.5 μm) have been implicated in cardiac and vascular damage, including endothelial dysfunction and promotion of atherosclerotic lesions [21]. Large numbers of particles of this size were present in the unfiltered mixtures.

The cigarette filter reduced the number of particles, especially the larger particles. This filter was more effective when used after hot extraction, but the remaining particle burden remained too high for injection. Of course, cigarette filters are not designed for liquids. The morphine recovery from the cigarette filters was nearly complete (90%) after two rinses. The unfiltered mixtures caused a block of the syringe filters, but the cigarette filtrate passed through them, as did the rinse volumes. Scott [18] found that both 0.22 μm and 0.45 μm syringe filters blocked with heroin injections, and abandoned them in favour of 5 μm filters. However, these blockages can be prevented by the use of a preliminary, coarse filter, such as the cigarette filter applied here.

The combination of cigarette filter then syringe filter mostly gave a good recovery of the extracted morphine. The 0.22 μm filter is considered to be sterilizing because, unlike the 0.45 μm filter, it will remove bacteria. In a trial with injecting drug users [22], it was found that 0.22 μm syringe filters were effective in removing bacteria from 3 out of 4 injections, while larger pore filters (15 - 20 μm) were completely inadequate.

## Conclusions

When a tablet of slow-release morphine (MS Contin^®^) is crushed and mixed with water, the resulting mixture contains millions of particles, of sizes from less than 5 μm to greater than 400 μm. These particles will cause great harm if injected into the bloodstream. The number of particles can be greatly reduced by filtration. A low-porosity syringe filter (0.45 or 0.22 μm) is most effective, but is likely to block unless a coarser filter is used first. Little of the morphine is lost in filtration if the filters are rinsed.

Hot extraction does not significantly increase extraction of morphine, and carries the risk of filtering a warm mixture which allows wax to pass through the filter, producing particles when it cools and solidifies. In practice, it is uncommon for solutions to be left for long before filtration and injection, producing the potential for a substantially greater level of filtrate contamination with wax than identified in the current study.

It is not possible to prepare an injection of pharmaceutical standard without clean facilities, as some particles will remain even after filtration through a syringe filter, and the injection will not be sterile. Also, the manufacturer cautions against using Millex^® ^sterile filters for suspensions or emulsions, because they are not designed for this purpose (Millipore Millex User Guide, 2008). However, filtration with a 0.45 μm or 0.22 μm filter can remove virtually all of the tablet-derived particles and should be a standard method of harm reduction for injecting drug users (a plain language summary of this study is provided in Additional file 1 in order to facilitate health interventions). The 0.22 μm filter is to be preferred, as it can remove the organisms (e.g. *Staphylococcus aureus, Candida*) which commonly produce cutaneous and systemic infections in injecting drug users [7,8,10]. Although it cannot remove the much smaller virus particles, including Hepatitis C [23], viral infections are mostly due to blood contamination from shared equipment and are avoided by not sharing. In one Canadian hospital, the two most common reasons for admission of injecting drug users were pneumonia and soft-tissue infections [24], both potentially preventable by effective skin swabbing and filtration of injections. The average daily cost of hospitalization was $CAN610 ($USD420 at the time of the study), which makes the use of alcohol swabs (currently <$USD0.02 in Australia) and filters (<$USD0.90) extremely cost-effective.

## Competing interests

The authors declare that they have no competing interests.

## Authors' contributions

SM contributed to the design of the study, carried out the particle counting, and drafted the manuscript. RB conceived of the study, participated in its design and coordination and helped to draft the manuscript. SB developed and conducted the morphine assays. BG interviewed the injecting drug users and summarised their methods. All authors read and approved the final manuscript.

## Supplementary Material

Additional file 1 **Plain Language Summary: **Effect of filtration on morphine and particle content of injections prepared from slow-release oral morphine tablets. Click here for file
