# Supplementary material for: Effect of filtration on morphine and particle content of injections prepared from slow-release oral morphine tablets
Source: Harm Reduct J. 2009 Dec 22;6:37. doi: 10.1186/1477-7517-6-37 (PMC2803777; doi:10.1186/1477-7517-6-37)
Supplement: Additional file 1 — Plain Language Summary: Effect of filtration on morphine and particle content of injections prepared from slow-release oral morphine tablets. [file 1477-7517-6-37-S1.DOCX]

**PLAIN LANGUAGE SUMMARY**

**Effect of filtration on morphine and particle content of injections prepared from slow-release oral morphine tablets**

*Raimondo Bruno, Stuart McLean, Susan Brandon & Barbara de Graaff*

**Reason for the study**

Many people,worldwide, inject solutions of drugs, that have been prepared from pharmaceutical tablets, such as morphine. Tablets not only contain the active drug, but also other inactive components, such as talc, cornstarch and wax. These components are included in order to form the tablet, and can also be used to slow the release of drug in order to prolong its effects. Many of these components do not dissolve in water. If these are injected into a blood vessel, undissolved particles of these inactive components will float downstream until they encounter a vessel too small to pass where they can become stuck, blocking blood supply – and causing tissue death - to any areas supplied by that vessel. Even particles as small as 10 micrometers (about one tenth of the width of a human hair, and difficult to see with the naked eye) can block the smallest blood vessels in the body and cause harm. Larger particles block larger vessels and are correspondingly more harmful.

We wanted to know how people filter morphine for injection, whether these methods are useful in removing particles, and whether they affect the amount of morphine received.

**What the study involved**

Tablets of morphine (MS Contin®) are commonly used by injecting drug consumers in the area where the study was conducted. We surveyed consumers about the types of filters that they used and interviewed consumers about how they prepare these drugs for injection, so that we could replicate their methods in a laboratory. People typically prepared tablets by removing the tablet coating, crushing them and stirring the powder in cold or heated water. While many people did not filter at all, common filters included hand-rolling cigarette filters, cotton balls and commercial syringe filters (0.45 or 0.22 micrometers). Samples were prepared using hot and cold methods and before and after filtering. The dose of morphine in each sample was measured by liquid chromatography. The number and size of particles in each sample was determined by examining the solution under a microscope, using a technique that is similar to that used to count blood cells. Only a small part of each sample can be viewed under the microscope, and while these examinations were performed in triplicate, they only provide estimates of the number of particles that would be in an injection of the drug.

**The key findings**

- Even injections prepared without a tablet, only using sterile water (‘control’ injections) contained many particles large enough to block blood vessels. These come from dust and contamination from the local environment, and are not usually visible to the eye.
- Unfiltered solutions of morphine were cloudy with visible particles and were estimated to contain millions of particles large enough to block blood vessels.
- Morphine content was largely unaffected by any filtering technique, provided that the filters were rinsed with small amounts of extra sterile water to recover any solution held up in the filters.
- Cigarette filters and cotton balls produced cloudy solutions. Cigarette filters removed most of the very large particles but had limited effect on smaller particles.
- Commercial syringe filters tended to produce solutions that looked clear to the eye but they blocked easily. If solutions were first filtered using a cigarette filter, and then filtered again by a commercial syringe filter, virtually all of the particles from the tablet were removed. If the filters were rinsed with a small amount of sterile water at each stage, there was no change in the amount of morphine recovered by this two-step filtering process. While the solution looked perfectly clear there are still some particles large enough to cause harms, but this is similar to the level seen in the control (sterile water only) solutions.
- Preparing the tablets by heating them produced a new set of problems – some of the waxy components of the tablet would melt due to the heat, and could be passed through the filter if the solution was even slightly warm. When the solution cooled, these waxy parts re-solidify and are liable to cause harm.
- Using too little sterile water to produce the solution can also cause morphine to precipitate, forming microscopic cystalline rods, which can also cause harms if injected. At this dose of morphine (60mg), this might occur when 3mL or less of sterile water was used

**Recommendations**

Injection of solutions derived from crushed tablets of morphine can cause great harm as each injection contains millions of particles large enough to block blood vessels.

Syringe filters (0.45 or 0.22 micrometers) can remove the vast majority of these particles, but they tend to block easily unless a coarse filter, like a hand-rolling cigarette filter, is used first. If the filters are rinsed with a small amount of sterile water at each stage, very little of the morphine is lost in this process.

If crushed tablets are mixed in water and heated, this can cause additional problems as an even slightly warm solution will allow wax to pass through filters, which on cooling will produce particles. Because of this risk, heating should be avoided. Furthermore, hot preparations do not contain more morphine than preparing injections without heat.

None of the filtering techniques could produce perfectly particle free solutions that would achieve the medical standard required to be considered safe. However, the harms from injection of these tablets are substantially reduced by a combination of an initial coarse filter and a syringe filter. The finest syringe filers (0.22 micrometers) are preferable as they can also remove bacteria that cause infections around injection sites and internally. Skin preparation with an alcohol swab is also essential to minimise the infection risk.
